# The use of rh-NGF in the management of neurotrophic keratopathy

**DOI:** 10.3389/fopht.2024.1408587

**Published:** 2024-07-08

**Authors:** Anna M. Roszkowska, Rosaria Spinella, Alessandro Calderone, Marianna Sindoni, Bogumił H. Wowra, Maciej Kozak, Katarzyna Sajak-Hydzik, Jorge Aliò

**Affiliations:** ^1^ Ophthalmology Clinic, Department of Biomedical Sciences, University Hospital of Messina, Messina, Italy; ^2^ Ophthalmology Department, Faculty of Medicine and Health Sciences, Andrzej Frycz Modrzewski Krakow University, Kraków, Poland; ^3^ Ophthalmology Unit, Department of Surgery, University Hospital of Messina, Messina, Italy; ^4^ Chair and Clinical Department of Ophthalmology, Faculty of Medical Sciences in Zabrze, Medical University of Silesia, Katowice, Poland; ^5^ Ophthalmology Department, Miguel Hernandez University, Alicante, Spain

**Keywords:** rh-NGF, cenegermin, neurotrophic keratitis, persistent epithelial defects, nerve growth factors, corneal sensitivity

## Abstract

Neurotrophic keratitis or keratopathy (NK) is a degenerative corneal disease induced by impairment of the trigeminal nerve function. This condition may lead to persistent epithelial defects, corneal ulceration, and perforation. The diagnosis of NK requires a careful investigation of any ocular and systemic condition associated with the disease and ocular surface and corneal sensitivity examinations. In the past, several medical and surgical procedures were used to treat this condition with different clinical effectiveness. Cenegermin is a recombinant human nerve growth factor (rh-NGF) that supports corneal reinnervation. Different clinical trials have demonstrated the safety and efficacy of topical cenegermin in patients with moderate to severe neurotrophic keratitis. In this review, we report the literature on clinical results regarding the treatment of NK with cenegermin since its approval by the European Medicines Agency (EMA) and the Food and Drug Administration (FDA) in 2017 and 2018, respectively.

## 1 Introduction

The extremely rich corneal innervation originates from the ophthalmic branch of the trigeminal nerve and controls the maintenance of ocular surface homeostasis, ensuring proper functioning of the ocular surface unit and corneal transparency, mandatory for visual performance (1). This function is exerted by promoting the release of neurotrophic factors such as nerve growth factor (NGF), brain-derived neurotrophic factor (BDNF), and neurotrophins 3 and 4 (NT-3 and NT-4) (2–6). NGF controls corneal healing and remodeling through the regulation of epithelial and stromal interaction (7, 8). A reduced level of NGF determines a nerve function impairment and altered corneal function with consequent visual loss (9, 10). Neurotrophic keratitis (NK) is a degenerative corneal disease resulting from severe impairment of the trigeminal nerve function, causing the deficiency of neural factors and the consequent loss of corneal tropism and impaired corneal healing (9). Nerve malfunction is the hallmark of NK, which manifests clinically in the early stage with epithelial defects that do not heal (10). Several factors have been identified as a possible cause of NK such as local or systemic diseases and surgeries, and traumatic, infective, and metabolic injuries affecting both the trigeminal and corneal nerves, with herpetic keratitis being the most frequently encountered (9, 11, 12). The disease may present with punctate epitheliopathy, persistent epithelial defects, corneal ulceration, and perforation and is accompanied by different grades of impaired corneal sensation (13). NK is classified as mild, involving only slight epithelial changes; moderate, with persisting epithelial defects (PEDs); and severe, with different grades of stromal involvement such as ulcer, corneal melting, and perforation (9, 13, 14). Recently, an additional classification based on Anterior Chamber Optical Coherence Tomography (AC-OCT) and *in vivo* confocal microscopy (IVCM) findings was proposed (15). The diagnosis of NK requires careful investigation of any ocular and systemic condition associated with corneal changes, and the testing of corneal sensitivity detecting hypoesthesia is considered as a main finding necessary to confirm diagnosis (13, 14). The therapy is challenging, and different therapeutic approaches, both medical and surgical, were adopted. In relation to NK severity, different therapies were used such as the therapeutic contact lens (CL), preservative-free artificial tears, topical autologous serum, Regenerating matrix agent (RGTA)—a matrix agent mimicking heparan sulfate, thyroxin beta-4, topical substance P, insulin-like GF1, amniotic membrane, conjunctival flap, tarsorrhaphy, and neurotization (16–18). Nevertheless, none of these therapeutic options provided satisfying results (14, 19, 20). Lambiase et al. demonstrated the effectiveness of murine NGF in promoting corneal healing and sensation recovery in patients with NK (21). Successively, the human recombinant NGF was used, and its efficacy in NK was demonstrated in the REPARO 1 and 2 studies (22, 23). The efficacy of rh-NGF was recently confirmed by Pflugfelder et al. in a multicenter randomized vehicle-controlled pivotal trial conducted in the United States (24). The rh-NGF 0.002% (cenegermin) was approved by the European Medicines Agency (EMA) in 2017 and the Food and Drug Administration (FDA) in 2018, and it actually represents the first-ever topical biologic drug for the first-line treatment of patients with NK stages 2 and 3 who do not respond to conventional treatment. In this review, we present the results of the clinical use of rh-NGF cenegermin (Oxervate; Dompé Farmaceutici SpA, Milan, Italy) in the treatment of moderate and severe NK.

## 2 Natural history of NK

The first recognizable alteration in the natural history of the pathology is the loss of corneal sensitivity. This factor generates a vicious cycle that induces and fuels the suffering of the cornea itself. The tear film is reduced in quantity and is unstable, the composition of its layers is altered, and the triggering stimulus mediates the production of proinflammatory cytokines and other harmful factors that change the homeostasis of the ocular surface and its ability to protect itself from external stimuli. The supply of growth factors and molecules useful to the limbal cells for the normal replacement of corneal cellular elements is greatly reduced, which remain senescent in the central portion of the cornea, thus worsening the quality of the eyelid redistribution mechanism. Inflammatory factors and aged dysfunctional cells accumulate, reducing the trophic and protective action of the tear film. As previously mentioned, the central lesion presents rolled borders consisting of elder cells that cannot link properly with their basal membrane, due to inflammation factors, mucin alterations, and the lack of proper tear film action. The chronicity of this process leads to the dysfunction of the intercellular junctions, worsening the corneal structural stability and inducing the risk of microbial overlap. The loss of stability of the tear film, the accumulation of inflammatory mediators, and the delay in the replacement of cellular elements establish the basis for the formation of the chronic epithelial defect. The stable epithelial defect leads to suffering of the stroma, exposed to the uncontrolled action of metalloproteinases, and therefore, to the possibility of melting and perforation, if the process is not promptly interrupted (25, 26).

## 3 Diagnosis

The diagnostic moment for a presumed patient suffering from NK cannot ignore the evaluation of his clinical history and general anamnesis. The slit lamp biomicroscopic examination is accompanied by various tests that contribute to the formulation of a certain diagnosis and the recognition of the various etiological and developmental factors. The analysis of the symptomatic process reported by the patient must consider the possibility that the symptoms vary considerably during the natural history of the pathology. The severity of the symptoms often does not correspond precisely to the quantity and quality of the recognizable signs, and therefore, to the progress of the pathology.

The symptoms present in the early stages are mainly linked to alterations of the ocular surface and therefore mainly consist of dryness linked to changes in epithelial homeostasis. The patient may experience photophobia and worsening of visual quality especially in the morning, if subjected to trigger situations caused by air conditioning, the prolonged use of electronic devices, and staying in dry and heated environments. The symptoms may decrease as the patient’s clinical condition worsens due to the reduction in corneal sensitivity. However, as soon as the central portion of the cornea is significantly affected, the patient will complain of a sharp decline in visual quality. In the early phase, patients complain of dry eye symptoms detectable with a short break-up time, a narrowed tear meniscus, and often, a superficial punctate keratitis with epithelial irregularities; in particular, in post-refractive surgery patients, the epithelial defect is recognizable in the central portion of the cornea, gradually forming coalescing erosions due to the failure of tissue healing and turnover mechanisms. The repetition of the damage and healing phases over time leads to the development of a permanent epithelial defect with blunt edges and a weak bond with the underlying Bowman’s membrane, easily recognizable with fluorescein staining, which leaks under the epithelium. The underlying stroma is exposed to harmful inflammatory factors, and even if initially sterile, the lesion can develop a secondary infection and perforate. In this phase, evaluating the presence of signs of distress such as Descemet’s folds, corneal vascularization, and cellularity or signs of inflammation in AC is mandatory. The presence of accessory signs can help in recognizing the noxa that led to the development of the pathology; *vice versa*, classifying the patient by their anamnesis is useful to differentiate peculiar signs of the different presentations of the disease.

Underlying signs of pathologies, such as outcomes of uveitis or keratitis, dystrophies, or corneal scarring, can be differentiated by the signs that suggest a systemic condition.

Corneal sensitivity is assessed by estimating the response of the cornea to an external stimulus and comparing the reaction during stimulation of the two eyes. In patients with NK, the response to stimuli and blinking are significantly reduced, as confirmed by the Cochet–Bonnet esthesiometer.

As has been widely demonstrated, the latest generation of instruments in the possession of ophthalmologists is an obligatory step for the study and follow-up of patients suffering from the different corneal pathologies (27). AS-OCT is useful to study corneal thickness changes in stages 2–3 of NK and is used to measure the depth of corneal ulcerations and stromal thickness, facilitating the diagnosis and improving the follow-up analysis (27, 28).

Analysis of nerve fibers can be performed with IVCM that allows qualitative and quantitative assessment in terms of density, morphology, course, and dimensions of corneal nerves. It has recently been introduced in clinical examination to study the cornea at the cellular level (29). As the nerve’s examination permits the detection of early changes occurring in peripheral neuropathies, the IVCM becomes, over time, an important tool for their assessment and follow-up (29).

Typical NK alterations visible on confocal microscopy are a decrease in corneal subbasal nerve fiber number and density, increased tortuosity of corneal nerves, a decrease in corneal epithelial and endothelial cell density, and an increase in the number of hyper-reflective keratocytes. In NK, IVCM is an important instrument to help in the diagnosis and to find the etiology of the disease, thanks to the accurate qualitative and quantitative information about subbasal and stromal nerves.

## 4 Clinical studies on the cenegermin treatment of NK

Cenegermin is a recombinant form of human nerve growth factor (rh-NGF) produced in *Escherichia coli*; its molecule is identical to human NGF. A preclinical study on different doses of human rh-NGF eye drops demonstrated the good tolerability and safety of this drug. Clinical studies such as REPARO1 and 2 demonstrated the clinical efficacy and safety of human recombinant NGF (rh-NGF) at doses of 10 μg/mL and 20 μg/mL in promoting corneal healing in mild and severe NK with a clinically better outcome with the higher concentration formulation without changes in tolerability.

The rh-NGF cenegermin was approved by the FDA at a concentration of 0.002%; Oxervate™ (Dompé Farmaceutici SpA, Milan, Italy), which is an ophthalmic solution of rh-NGF, is the first-ever topical biologic drug approved in the field of ophthalmology and the first drug approved for treatment of NK (30).

It was demonstrated that the drug has high specificity for the anterior segment of the eye, playing an important role in supporting corneal healing and maintaining corneal integrity with the posology of one drop × six times/day × 8 weeks.

The studies and trials recorded so far have highlighted that most adults affected by moderate or severe NK experienced complete corneal healing after up to 8 weeks of topical cenegermin therapy (31).

In this review, we analyze the results of 15 studies presented in the literature database, reporting the clinical outcome of the therapy of NK with rh-NGF. Eight papers report results in adult patients and seven papers report results in a pediatric population.

**Tables 1**, **2** show the studies on adults and pediatric patients, respectively.

**Table 1:** Clinical outcomes in adult patients with NK treated with cenegermin.

Study	NK etiology	Patients and methods	Results
Pflugfelder et al. (32)	HSV keratitis, ocular surgery, and dry eye disease	48 patients Cenegermin vs. vehicle Follow-up was 24 weeks	Vehicle 29.2% healed Cenegermin 69.6% healed
Sacchetti et al. (33)	HSV keratitis, post-surgical trigeminal damage, post-ocular surgery, severe dry eye disease, diabetes, ocular caustication, and ocular cicatricial pemphigoid Diabetes, high blood pressure, and atopic diseases	38 patients Cenegermin vs. AMT 12 months of follow-up	At 2 months Cenegermin 96% healed AMT 86% healed Recurrence during 12 months: AMT 46% Cenegermin 13%
Garciía-Delpech et al. (34)	HSV keratitis or keratouveitis, secondary to radiation therapy of a parotid gland tumor, idiopathic	5 patients 4 years follow-up	No recurrence
Roszkowska et al. (35)	HSV keratitis, post-neuroma surgery diabetes, previous ocular surgery, Graves’ disease, previous trauma, rheumatoid arthritis, and atopic conjunctivitis	21 patients Follow-up of 8 weeks	PED healed in 37.5% of eyes after 4 weeks 100% of eyes at 8 weeks Ulcers healed in 69% of eyes at 4 weeks 100% of eyes at 8 weeks
Pedrotti et al. (36)	HSV keratitis, PKs, trauma, exposure keratopathy, and acoustic nerve surgery	18 patients Follow-up of 8 weeks and 4 and 8 months	Complete corneal healing was observed in 78% of patients at 8 weeks 83% at 4 months 72% at 8 months In three patients, the treatment led only to a reduction in the epithelial defect but failed to completely resolve it.
Di Zazzo et al. (37)	HSV keratitis and recurrent epithelial and stromal immune keratitis	3 patients	Complete corneal healing was achieved after a mean of 47 ± 12 days, with all ulcers repairing within the standard 8-week cenegermin treatment. Results stable, with no recurrence of epithelial defects.
Mastropasqua et al. (38)	HSV keratitis, keratoplasty, chemical trigemin-lysis, and diabetes	8 patients Follow-up of 8 weeks	Complete corneal epithelial healing for 83.3% after 4 weeks 100% after 8 weeks Significant improvement of SBNP inn IVCM
Hao et al. (39)	Trigeminal nerve injury caused in a patient with previous keratoplasty, dry eye, scleroderma, keratoplasty in a type 2 DM patient, and chemical burn	6 patients/9 eyes Follow-up of 8 weeks	Complete recovery within 8 weeks Significant increase of CNFD in IVCM

**Table 2:** Clinical outcomes in pediatric patients with NK treated with cenegermin.

Study	NK etiology	Materials and methods	Results
Fausto et al. (40)	Bilateral NK in pontine tegmental cap dysplasia with bilateral cranial nerves VI and VIII palsies	Case report 9-year-old patient Follow-up of 6 months	Right eye At week 4, reduction of the corneal opacity Corneal sensitivity improvement After 8 weeks, reduction of corneal neovascularization and opacity 6 months stability Left eye Resolution of the superficial punctate keratopathy and improvement in corneal sensitivity
Hatcher et al. (41)	Hereditary NK Surgery, trauma, and infection	8 patients/9 eyes Follow-up of 10 months	63% of patients completed the full 8-week therapy course with clinical improvement, with a mean recurrence-free period of 10 months
Pedrotti et al. (42)	Surgery for rhabdomyosarcoma and radiotherapy	Case report 3-year-old boy Follow-up of 8 weeks	Complete healing of the corneal epithelium was achieved after 3 weeks
Elhusseiny et al. (43)	Surgery or radiotherapy for intracranial mass Stuve–Weidemann syndrome	4 pediatric patients The mean follow-up time was 15 ± 9.6 months.	After 8 weeks Marked improvement in epitheliopathy in all patients. In two patients, small epithelial defects at the last follow-up persisted, with variable improvement of the NK.
Leto et al. (44)	Bilateral NK in a child affected by Goldenhar syndrome with absence and hypoplasia of the right and left trigeminal nerves	Case report 16 months	Complete healing was achieved in both eyes after treatment. No recurrence Progressive reduction of opacities during the follow-up period
Papadopoulos et al. (45)	Ocular infection	Case report 9-year-old patient Follow-up of 8 weeks	Complete restoration of corneal sensitivity and an increase in visual acuity were achieved.
Niruthirsard et al. (46)	Neurosurgery for synchronous brain tumors	Case report 4-year-old boy Follow-up of 2 years	At the end of the therapy, corneal epitheliopathy was markedly improved and central corneal sensation was normalized and maintained during the follow-up period. IVCM demonstrated the presence of subbasal corneal innervation.

### 4.1 The studies on adult patients

A multicenter randomized double-masked study by Pflugferlder et al. on 48 patients with NK that compared cenegermin 0.002% to vehicle eye drops showed significantly higher healing rate in the rh-NGF group. The authors demonstrated that cenegermin-treated patients showed statistically significant reductions in lesion size and disease progression rates during masked treatment, and no severe adverse effects were experienced (32).

Sacchetti et al. compared the outcomes of 38 patients with NK treated with amniotic membrane transplantation (AMT) to those treated with cenegermin eye drops. At the end of treatment (2 months), a complete healing was observed in 86% of AMT-treated patients and in 96% of cenegermin-treated patients. During 12 months of follow-up, recurrence occurred in 46% of patients treated with AMT and in 13% of patients treated with cenegermin. The study confirmed the effectiveness of both AMT and cenegermin in stages 2 and 3 of NK; however, the last one was associated with minor frequency of recurrences and a high satisfaction of patients (33).

In a case series study, Garcia-Delpech et al. demonstrated the effectiveness of cenegermin eye drops in five adult patients affected by severe and moderate NK at stage 2 or 3 after showing poor results of the conventional therapy. Treatment was well tolerated by patients, and a complete corneal healing with a trend improvement in visual acuity (VA) and sensitivity was observed. In this study, during 4 years of follow-up, no recurrence of NK was observed (34).

Roszkowska et al. examined 21 patients affected by moderate and severe NK treated with cenegermin. Participants were evaluated at baseline and after 4 and 8 weeks of protocol therapy. In the moderate NK group at 4 weeks, PEDs healed in 37.5% of eyes, and at 8 weeks, a complete recovery was registered in 100% of eyes. In contrast, in the severe NK group, at 4 weeks of treatment, 69% of ulcers were closed, while at 8 weeks, 100% of eyes were completely healed.

This study showed that eyes affected by severe NK had better healing than eyes with moderate NK, but such finding needs to be further investigated (35).

Pedrotti et al. made an 8-month follow-up of NK that correlated with various systemic diseases (such as burns or acoustic nerve surgery with trigeminal damage) after treatment with cenegermin eye drops. Eighteen eyes of 18 patients were studied at baseline at 8 weeks (end of therapy) and at 2, 4, and 8 months after treatment (follow-up) (35). At 8 weeks in 78% of patients, complete healing was observed, and in 78%, 83%, and 72% of patients, respectively, at 2, 4, and 8 months of follow-up, resolution of signs and symptoms was observed (36).

Di Zazzo et al. studied three patients affected by NK stage 2 or stage 3 with a unilateral lesion not responding to classic medical therapies. Patients were evaluated at baseline, then weekly for 8 weeks and monthly thereafter. Complete corneal healing was achieved after a mean of 47 ± 12 days, with all ulcers repaired within the standard 8-week cenegermin treatment. The three eyes maintained a stable, healed corneal epithelium during the follow-up period with no recurrence of epithelial defects (37).

Mastropasqua et al. examined 18 patients affected by various NK etiologies who were treated with topical antibiotics, lubricants, and therapeutic CLs before enrolling in the study. After 4 weeks of cenegermin eye drops, 83.3% of patients showed complete corneal healing. The Schirmer I test showed a significant increase in tear film secretion after 8 weeks of treatment compared to baseline, and the Cochet–Bonnet esthesiometry demonstrated an increase in corneal sensation as compared to baseline. Additionally, the IVCM evaluation of the subbasal nerve fibers showed an increase in the diameter and number of nerve fibers as compared to the baseline values (38).

Hao et al. evaluated nine eyes of six patients with NK at stages 2–3 not responding to previous medical therapy. After 8 weeks of cenegermin treatment, there was a significant difference in corneal fluorescence staining scores, and the epithelial defects recovered completely. Based on confocal microscopy findings, the corneal subbasal nerve fiber density and number increased (39).

### 4.2 The studies on pediatric patients

In a pediatric population, cenegermin was administrated in persistent visually threatening cases that did not respond to conventional treatments. In the literature, only two articles report a case series of four and eight patients, with the other articles being single case reports with severe underlying diseases.

Fausto et al. reported a case of a 9-year-old patient affected by a pontine tegmental cap dysplasia with bilateral cranial nerves VI and VIII palsies that caused a stage 2 NK in the right eye and a stage 1 NK in the left eye. Cenegermin was administered in both eyes according to the therapy protocol. At week 4, the corneal opacity of the right eye was reduced, and sensitivity was improved. At week 8, the corneal neovascularization was markedly reduced, and the epithelium seemed normal. Over the next 6 months, the opacity decreased in size and density and only a fine neovascularization remained. In the left eye, there was a complete resolution of the superficial punctate keratopathy and an improvement of sensitivity (40). Topical cenegermin was well tolerated and no side effects were reported.

Hatcher et al., in a retrospective case series on eight pediatric patients with acquired and hereditary NK, showed a clinical improvement of corneal defects and best-corrected visual acuity in five patients. These improvements persisted through the 10 months of follow-up. Side effects reported during therapy were ocular pain, difficulty sleeping, and persistent corneal thinning. Results of this study permitted us to understand that cenegermin gives a modest support for NK in the pediatric population and that the primary benefit was an improvement in corneal epithelial stability (41).

Pedrotti et al. reported the use of cenegermin in a 3-year-old child with NK after surgery for rhabdomyosarcoma of the jaw. A gradual improvement of corneal signs was noted after 2 weeks, and on the third week, the corneal epithelium was reestablished (42).

Elhusseiny et al. evaluated the efficacy of cenegermin drops in a retrospective study on four pediatric patients after failure of the classic medical therapy. In all cases, at the end of treatment, a marked improvement of the epitheliopathy was demonstrated with a general improvement of corneal signs and symptoms (43).

Leto et al. evaluated a bilateral NK in a pediatric patient with Goldenhar syndrome associated with hypoplasia of both trigeminal nerves. After 8 weeks of therapy, a complete healing in both eyes was achieved, and during the 16 months of follow-up, no complications or regression of the disease was detected, whereas corneal opacity became clearer, and there was no significant improvement of corneal sensation (44).

Papadopoulos et al. showed a complete restoration of the corneal surface and an increase in visual acuity after cenegermin administration in a 9-year-old child with NK refractory to usual therapy (45).

Niruthisard et al. reported a case of a 4-year-old child affected by severe NK, lagophthalmos, and facial palsy following neurosurgery for synchronous brain tumors initially treated with topical antibiotics, topical steroids, artificial tears, and lateral tarsorrhaphy with a transient result. After cenegermin treatment, the central corneal sensitivity recovered and the corneal epitheliopathy was significantly improved with a positive outcome lasting for 2 years after therapy (46).

## 5 Discussion

NK is a sight-threatening condition due to the pathology of the trigeminal nerve and its ophthalmic branch terminations. Corneal alterations characterize the moderate and severe forms with significant reduction of the corneal sensation and loss of corneal trophism. Cenegermin is the first topical biologic drug for first-line treatment in patients with NK stages 2 and 3. The data presented in the literature demonstrate the clinical efficacy and safety of the treatment. Correct indications and prompt treatment with rh-NGF promote healing of persistent epithelial defects and induce healing of ulcers and recovery of the corneal sensitivity. The restoration of the corneal thickness and the healing of PED and ulcers after the treatment of rh-NGF confirm the crucial role of NGF in promoting the release of factors involved in the maintenance of the integrity of the corneal epithelium and ocular surface hemostasis. The overall reported results might consider the use of cenegermin eye drops in all cases of severe and moderate NK if the conventional therapy proved to be ineffective. In addition, unlike surgical treatments, cenegermin did not cause relevant side effects, thus having no impact on the patient’s quality of life.

The analyzed studies show the safety and prolonged effect of cenegermin in the treatment of both adult and pediatric NK with recovery in almost all patients. Nevertheless, the efficacy of the treatment and its stability have been proven in pediatric cases; such data need further investigation using prospective, randomized clinical trials with standardization of evaluation procedures and assessment on corneal sensitivity and IVCM.

The high cost of the drug and the need for cold chain storage and delivery limit its wide use.

## Author contributions

AR: Conceptualization, Writing – review & editing. RS: Writing – review & editing. AA: Writing – review & editing. MS: Writing – review & editing. BW: Formal analysis, Writing – review & editing. MK: Writing – review & editing. KS-H: Writing – review & editing. JA: Writing – review & editing.
